# Bayesian meta-analysis now – let's do it

**DOI:** 10.3325/cmj.2020.61.564

**Published:** 2020-12

**Authors:** Branimir K. Hackenberger

**Affiliations:** Department of Biology, Josip Juraj Strossmayer University, Osijek, Croatia *hackenberger@biologija.unios.hr*

Meta-analysis is a statistical tool that allows the analysis of results from various scientific studies, which are often not performed in the same place or using the same method. The data used in meta-analysis may be proprietary or may be obtained from literature or various databases. Meta-analysis is a crucial part of many systematic reviews, although not all systematic reviews include a meta-analysis. Therefore, meta-analysis should not be equated with systematic reviews. It is not incorrect to say that meta-analysis synthesizes the results from several studies and yields a new set of results. In other words, meta-analysis itself can, under certain conditions, be considered a method of producing new data. There are two basic models of meta-analysis, the fixed-effects and random-effects model. If there is no statistical or methodological heterogeneity and if it is not necessary to generalize the conclusions, a fixed-effect model is applied. On the other hand, if a generalized conclusion is to be obtained and if results from more than five different studies are taken into account, a randomized-effects model is appropriated. While the fixed-effect model assumes the existence of a common fixed parameter in all studies, the random-effects model assumes that such a common fixed parameter does not exist. Therefore, the fixed-effects meta-analysis model's total effect is an estimator of the combined effect of all studies. In contrast, the random-effect meta-analysis's full effect is an estimator of the mean value of the true effect distribution.

The number of biomedical and clinical trials using meta-analysis has increased exponentially (Figure 1). The number of studies that used a completely different approach to meta-analysis, ie, Bayesian meta-analysis, is also growing. In general, we can argue that Bayesian statistics provides an increasing support to research results interpretation. Although by reading various popular scientific articles, and even some letters to editors, and listening to discussions at conferences, one gets the impression of a conflict between frequentist and Bayesian statistics users, this is far from true. These are just two different approaches, both of which, under characteristic conditions, can give useful results. The fundamental difference between Bayesian meta-analysis and frequentist meta-analysis is that Bayesian meta-analysis treats both data and model parameters as random variables. The primary task of Bayesian statistics is to determine the probability function of obtaining data depending on the given, ie, predicted parameters, where the parameters are treated as random variables. It is exciting and sometimes extremely useful that we can use subjective beliefs to construct the parameter distribution in addition to the results of the meta-analysis. This fact is often pointed out by the critics of Bayesian statistics, who call this type of statistics “subjectivist statistics.” The constructed joint prior probability density parameter function (attached to possible results) is combined with the likelihood function (attached to hypothesis) to obtain a joint posterior probability density function. While the probability density function shows how likely it is that specific data points appear, the likelihood function represents the likeliness of different distribution parameters. The Bayesian meta-analysis should be understood as an opportunity to get a more accurate meta-analysis.

**Figure 1:**
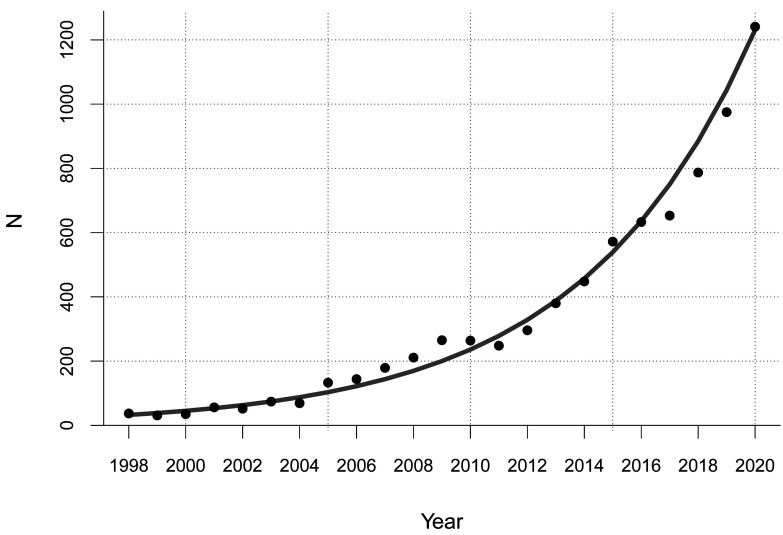
Number of published papers over the years (contained in the sciencedirect.com database) in which Bayesian meta-analysis was used.

New versions of most of the commercial statistics software increasingly contain tools that enable the implementation of Bayesian statistics. However, we can run Bayesian statistics very successfully on open source platforms such as Python or the R programming environment. Three Bayesian statistics-specific software tools (OpenBUGS, JAGS, and Stan) can be used seamlessly within the R environment. Stan might be the optimal solution because it is very well documented, relatively frequently updated, and uses Markov chain Monte Carlo (MCMC) and Hamiltonian or hybrid Monte Carlo (HMC) algorithms for sampling from probability functions. Python users can benefit significantly from the *PyMare* package (Python Meta-Analysis and Regression Engine) (1). Several excellent R packages contain the full range of functions required to perform Bayesian meta-analysis in the R environment. The functions of *metaBMA* package enable the computation of the probabilities of the posterior model for standard meta-analysis models. These posterior probabilities could be used as the weighted average for random effect and fixed-effect models for estimating the overall mean effect size. This package allows us to define a wide range of priors for the mean effect size and the heterogeneity coefficient. Besides, meta-analysis of continuous and discrete moderators using precompiled Stan models can be fitted and tested. In this way, Bayes factors can be computed and Bayesian model performed averaging across meta-analysis with and without moderators (2). Another package containing functions for the Bayesian meta-analysis of diagnostic test databased on a scale mixtures bivariate random-effects model is the *bamdit* (3), while the *meta4diag* package includes Bayesian inference analysis for bivariate meta-analysis of diagnostic test studies using integrated nested Laplace approximation (INLA), a method for approximate Bayesian inference. In recent years, it has established itself as an alternative to other methods, such as the MCMC, due to its speed and ease of use through the R-INLA package (4).

Open-source software programs such as R, owing to comprehensive cooperation and strict internal review, boast rapid reacting to new achievements and a relatively high rate of error correction. Thus, the *NMADiagT* package for network meta-analysis of multiple diagnostic tests is based on the hierarchical summary receiver operating characteristic model developed in 2018 and the hierarchical model developed in 2019. These package functions enable performing meta-analysis for one to five diagnostic tests to simultaneously compare multiple tests within a missing data framework (5). *BayesCombo* package (6) combines diverse evidence across experiments using Bayes factors, based on a method developed by Kuiper et al (7). It is certainly worth mentioning the already standard package of *bmeta*, which can be a useful tool for beginners in Bayesian meta-analysis. This package includes functions for calculating various effect sizes or outcome measures for different data types based on MCMC simulation. It allows users to fit fixed-effects and random-effects models using different priors of the data. If the effects of other covariates are observed, meta-regression can be performed. The software package also provides functions for creating posterior distribution plots and forest plots to display the primary output model. It allows the use of traceplot and other diagnostic plots to evaluate the model's fit and performance (8). One of perhaps most comprehensive packages for Bayesian meta-analysis is *brms*. This package has a wide range of distributions and link functions, allowing users to fit linear, robust linear, count data, survival, response times, ordinal, zero-inflated, hurdle, and even self-defined mixture models, all in a multilevel context. Further modeling options include nonlinear and smooth terms, auto-correlation structures, censored data, meta-analytic standard errors, and quite a few more. Furthermore, it can be used to predict all parameters of the response distribution to perform distributive regression. Prior specifications are flexible and explicitly encourage users to apply prior distributions that reflect their beliefs. The fit model can be easily assessed and compared with posterior predictive checks and leave-one-out cross-validation (9,10). Of particular interest is the *jarbes* package, which provides a new class of Bayesian meta-analysis models named hierarchical meta-regression (HMR). HMR aims to incorporate the data collection process into meta-analysis, which results in a model addressing the internal and external validity bias. In this way, it is possible to combine different types of studies (11). Of course, many packages include functions that enable useful Bayesian meta-analysis. The appearance of new packages with new algorithms is also expected very soon after their publication.

## Bayesian meta-analysis in COVID-19 research

One of the earliest studies implementing Bayesian meta-analysis for the analysis of COVID-19 clinical trials was that by Javdani et al (12). The meta-analysis included five randomized controlled trials with a total of 591 patients treated by four different agents: arbidol, favipiravir, lopinavir-ritonavir, and hydroxychloroquine. Based on the surface under the cumulative ranking curve probabilities calculated for each treatment, the authors concluded that hydroxychloroquine had the highest treatment efficiency. Later, a random effects network meta-analysis by Siemienuk et al (13) showed that patients receiving corticosteroids had a lower risk of death and mechanical ventilation than those randomized to standard care. Using a similar method, Zhang et al (14) showed that, among 19 agents investigated, dexamethasone led to the lowest risk of mortality and mechanical ventilation compared with the standard of care. Fiolet et al (15) summarized the results of 25 observational studies, three randomized controlled trials, and one interventional non-randomized study on hydroxychloroquine treatment with or without azithromycin and the mortality of COVID-19 patients. The authors first performed a classical random-effect meta-analysis, then confirmed their findings using a Bayesian meta-analysis, concluding that hydroxychloroquine alone was not sufficient to treat patients diagnosed with COVID-19 and that combined treatment with hydroxychloroquine and azithromycin increased the mortality risk. Several studies have estimated the efficacy of infection control measures. A systematic review and meta-analysis by Chu et al (16), based on frequentist and Bayesian meta-analyses and random effects meta-regression, showed that physical distancing of 1 m or more and optimum use of face masks, respirators, and eye protection significantly contributed to the prevention of the transmission of SARS-CoV-2. Moreover (17), in a Bayesian network meta-analysis, Yin et al showed that continuous wearing of N95 respirators throughout the shift could serve as the best preventive measure for health care workers compared with other protective equipment. The Bayesian framework has also been implemented in the estimation of the secondary attack rate of COVID-19, ie, the probability of the disease spread in close-contact environments (eg, family, household, dormitory, etc), which can be influenced by many factors, including personal hygiene, social behaviors, and features of close-contact environments. Namely, Huang et al (18) estimated that the secondary attack rate of COVID-19 in Taiwan was 0.42%-1.69% at the time when their results were published and predicted that in the future it would range 0.08%-8.32%. Finally, a Bayesian meta-analysis has also been implemented for investigating the association of smoking status with SARS-CoV-2 infection and the course and outcome of the disease. Carmona-Bayonas et al (19) concluded that, similar to other respiratory disorders, active smoking increased the severity of COVID-19, while Simons et al (20) showed that current and former smokers, compared with never smokers, were at increased risk of hospitalization, increased disease severity, and mortality.

## Bayesian meta-analysis in other research

In the last ten years, Bayesian meta-analysis was predominantly implemented in medical research, neuroscience, and psychology. Still, we recognized the Bayesian approach's advantages in other fields, such as computer science, environmental science, social sciences, engineering, and economics. For example, Thompson and Semma (21) introduced Bayesian meta-analysis of data from adolescent development research. The authors assessed the impact of media literacy interventions on media literacy skills and attitudes, and risky health behaviors. Apart from demonstrating how to compute and interpret several meta-analytical quantities, they also discussed the advantages and disadvantages of the frequentist and Bayesian methods and provided the full R code used for their research. As an example from medical research, Charkos et al (22) used the Bayesian framework to conduct a meta-analysis of research on the association between thiazide diuretics and hip fracture risk. By analyzing 12 cohort studies including 2 537 871 participants, the authors showed that thiazide diuretics were associated with a lower risk of hip fracture, suggesting that these substances could have a significant role in protecting the general populations from hip fracture. Additionally, Fang et al (23) showed that fluconazole reduced the risk of the mycological cure rate in oral candidiasis better than other tested drugs.

Bayesian meta-analysis has also been implemented to evaluate the efficacy of non-pharmacological interventions on agitation in people with dementia. Based on data from 65 randomized controlled trials, Leng et al (24) confirmed that non-pharmacological interventions effectively ameliorate agitation in people with dementia and should be applied during routine care. In environmental sciences and agriculture, Bayesian approaches have been used to estimate the value of reducing eutrophication in marine areas in Europe (25), synthesize decay rate constant estimates for common fecal indicator bacteria (26), as well as assess the effects of fasting, transport, and lairage times on the attributes of pork meat quality (27), to name a few topics. The use of meta-analysis in ecology has been growing since the 1990s, and meta-analysis has been used to discern general patterns from data on various species and study sites. Due to the sizeable among-study variation in effect sizes, the Bayesian framework could be a promising tool for performing meta-analyses in the field. A review article by Pappalardo et al (28) compared the traditional and Bayesian approaches to ecological meta-analysis, concluding that using the frequentist and Bayesian framework should depend on the type of research and the quality and quantity of the available data.

As shown, Bayesian meta-analysis already has a diverse and practical application today. The main advantage of the method is appropriately taking into account the uncertainty around the heterogeneity variance. The frequentist approaches use the point estimate of the heterogeneity variance as a fixed quantity, which leads to variability underestimation. Furthermore, the Bayesian meta-analysis enables us to perform sensitivity analyses by changing distributional assumptions and incorporate *a priori* knowledge in the model. All this shows that Bayesian meta-analysis should not be considered as a competitive method of frequentist meta-analysis, but only as an additional tool that can help us achieve a much more reliable result. So let's go! Let the Bayesian approach enrich your next meta-analysis.
